# Comorbidity of the Mental and Cardiac Pathology in Patients of the Eastern Europe

**DOI:** 10.1192/j.eurpsy.2026.10442

**Published:** 2026-07-31

**Authors:** D. Labunskiy

**Affiliations:** Ogarev Mordovia State University, Saransk, Russian Federation

## Abstract

**Introduction:**

Comorbidity plays an important role both in mental and cardiac pathology.

**Objectives:**

To assess the significance of psychosocial factors, anxiety and depressive disorders in predicting the characteristics of comorbid conditions in patients with coronary heart disease (CHD).

**Methods:**

The study included 132 patients aged 37 to 66 years with a diagnosis of CHD and anxiety-depressive spectrum disorders. Depression was detected in 42% of patients, anxiety - in 25%; combined manifestations of anxiety, depression, along with asthenia, in 33%.

**Results:**

The total percentage of prediction (Concordant) in the general group of patients was 95.4%, the coefficient of association of features (Somers’D = 0.910); in the group of men - 95.5%, Somers’D=0.912; in the group of women - 93.1%, Somers’D=0.877. The predictors of the prognostic models with a high level of significance (p=0.0001) included known factors that determine cardiovascular risk (CVR): patient age, arterial hypertension, diabetes mellitus, dyslipidemia, left ventricular hypertrophy, cardiac arrhythmia,. A relationship was established between the age of onset of mental disorder and past traumatic events (p=0.0001). The relationship between stressful events preceding the development of anxiety and depressive disorders in the formation of predictors affecting the dynamics of the course and diagnosis of CHD is shown.

**Conclusions:**

. Using logistic regression, a high compatibility and relationship of CVR factors, psychosocial factors, anxiety and depressive disorders included in the list of significant predictors for predicting the characteristics of comorbid conditions and the progression of coronary heart disease was established. The results obtained are guidelines for an interdisciplinary approach to the correction and prevention of combined pathology.

**Disclosure of Interest:**

None Declared

